# PARP inhibitors induce a senescence phenotype in non‐small cell lung carcinoma cell lines

**DOI:** 10.1002/2211-5463.70206

**Published:** 2026-02-19

**Authors:** Camille Huart, Manon Van den Abbeel, Christoph Schifflers, Karim Bouhjar, Andrea Scarmelotto, Alexis Khelfi, Anne‐Catherine Wera, Carine Michiels

**Affiliations:** ^1^ Biochemistry and Cellular Biology Research Unit (URBC), Namur Research Institute for Life Sciences (NARILIS) University of Namur (UNamur) Belgium; ^2^ Centre de recherche du CHUM (CRCHUM) and Institut du cancer de Montréal (ICM), Université de Montréal Canada; ^3^ Namur Research Institute for Life Sciences (NARILIS), Laboratory of Analysis By Nuclear Reactions (LARN) University of Namur (UNamur) Belgium; ^4^ Molecular Physiology Research Unit (URPhyM), Namur Research Institute for Life Sciences (NARILIS) University of Namur (UNamur) Belgium; ^5^ Centre Hospitalier Universitaire CHU UCL Namur Service de Radiothérapie Belgium

**Keywords:** lung cancer, PARP inhibitors, senescence, senolytics

## Abstract

Several anticancer treatments have been shown to activate the DNA damage repair pathway but also, in some cases, to lead to therapy‐induced senescence. Senescent cells can either exert protumoral or antitumoral effects. However, it remains poorly characterized which treatments lead to a senescent state. Our findings identify Talazoparib, a PARP1 inhibitor, as the most potent inducer of senescence in nonsmall cell lung carcinoma cell lines among a variety of PARP1 inhibitors. In the absence of PARP1, no senescence phenotype was observed, thus demonstrating that PARP1 is necessary for the induction of senescence in nonsmall cell lung carcinoma cells exposed to Talazoparib. This enzyme is also required to induce an increase in cell death with the addition of Navitoclax (ABT‐263), a senolytic drug. As senescence has been shown to have several protumoral effects, these results demonstrate the importance of determining which anticancer therapies induce a senescence phenotype as it could lead to not only treatment failure but alsodrug combinations targeting this pathway to further enhance anticancer treatment efficacy.

## Graphical abstract

Talazoparib is the most potent inducer of senescence among different PARP1 inhibitors in human NSCLC cells. In the absence of PARP, no senescence phenotype was observed, demonstrating that PARP1 is necessary for the induction of senescence by this inhibitor. Additive effect to induce cell death was observed with Navitoclax, a senolytic drug, in a PARP‐dependent manner.

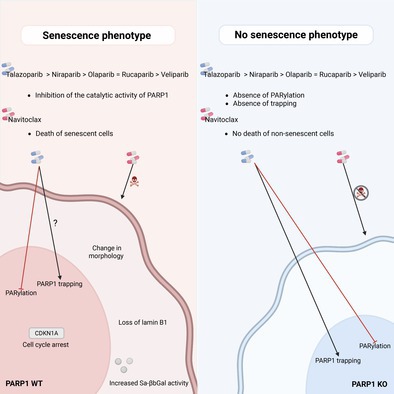

**Abbreviations**
DDR: DNA damage repair
NSCLC: non‐small cell lung carcinoma
PARP: poly‐ADP‐ribose polymerase
SA‐β‐Gal: senescence‐associated beta‐galactosidase

The poly‐ADP‐ribose polymerase (PARP) family is composed of 17 members [1]. Poly‐ADP‐ribose polymerase 1 (PARP1) is the most well‐known member of the PARP family. This enzyme is notably heavily involved in the DNA damage response (DDR). Upon genotoxic stress, PARP1 is recruited and uses nicotinamide adenine dinucleotide (NAD+) as a substrate to synthesize poly‐ADP‐ribose (PAR) polymers. PAR polymers attach to PARP1 itself and to multiple target proteins, serving as a platform to recruit, among others, the DDR machinery [2].

Due to the importance of PARP1 for the DDR to take place, several PARP inhibitors (PARPi) have been designed. Several of these PARPi are currently approved as anticancer agents due to their ability to induce ‘synthetic lethality’ in cancer patients harboring BRCA1/2 mutations [3]. PARPi are now being studied in many other solid tumors as single agents, either alone or in combination with chemotherapy and radiotherapy [4, 5]. PARPi inhibit PARP1 activity through NAD+ competition, thus preventing the formation of PAR polymers. Interestingly, even if PARP1 is often considered to be the major target of PARPi, some off‐target effects related to other PARP members such as PARP2 and PARP3 cannot be excluded as the structure of their NAD‐binding domain is similar [6].

Initially, it was thought that the cytotoxicity of the inhibitors came from the catalytic inhibition of PARP1. However, it has later been demonstrated that the cytotoxicity of these inhibitors mainly derives from their ability to trap PARP, mostly PARP1, onto the DNA [7]. In the absence of PARPi, the negative charge of the PAR polymers, which are present on PARP1 allows the release of PARP1 from the chromatin. In the presence of PARPi, the formation of PAR polymers is prevented, leading to the trapping of PARP1 onto the chromatin. The PARP‐DNA complexes are highly toxic as these complexes interfere with the DDR [7]. As aforementioned, PARPi are used to catalytically inhibit PARP1. However, these inhibitors can also affect other members of the PARP family, in particular PARP2 and PARP3. Talazoparib (BMN 673) has been described to be the most potent inhibitor of PARP1 so far [8]. Furthermore, it was also demonstrated that the various PARPi do not display the same ability to trap PARP onto the chromatin. The level of PARP1 trapping elicited by the inhibitors of PARP can be quantified in the following manner: Talazoparib > Niraparib > Olaparib ≈ Rucaparib > Veliparib [9].

Recent studies have demonstrated that anticancer treatments capable of activating the DDR can often lead to therapy‐induced senescence (TIS) [10]. PARPi, mainly Olaparib, has been shown to induce cellular senescence, which is regarded as a state of irreversible proliferation arrest maintained by p16/RB and/or p21/p53 [11, 12, 13, 14]. Senescent cells are characterized by several features *in vitro* and *in vivo*. Indeed, senescent cells display an enlarged shape; the integrity of the nucleus of the senescent cells is compromised due to a decrease in the expression of Lamin B1 (LMNB1) and an increased activity of senescence‐associated beta‐galactosidase (SA‐β‐Gal). Senescent cells also activate several prosurvival factors in order to resist apoptosis. Indeed, increased translation of anti‐apoptotic proteins, namely Bcl‐2 and Bcl‐XL, is observed [15, 16, 17]. Senescent cells exert several effects on surrounding cells mainly through a specific secretory phenotype, called the senescence‐associated secretory phenotype (SASP). The SASP is composed of cytokines, chemokines, proteinases, but also of nonprotein molecules, including reactive oxygen species (ROS), nitric oxide and related nitrogen species (NOx), microRNAs (miRNAs), and small molecules [18, 19]. Due to the emergence of new technologies such as single‐cell RNA‐sequencing and single‐nucleus RNA‐sequencing, it was discovered that cellular senescence is quite heterogeneous [20].

In the context of cancer, it was demonstrated that senescent cells can exert both protumoral and antitumoral effects [21, 22]. Baker et al. showed that the clearance of senescent cells (p16Ink4a‐positive cells) resulted in a reduced incidence of spontaneous tumorigenesis and cancer‐associated death in aged mice [23]. The interest in targeting senescent cells in the context of cancer further increased as cellular senescence was recently added as a Hallmark of Cancer [24]. Senescent cells can be specifically targeted by senolytics or senomorphics. Senolytics refer to the lysis of senescent cells, whereas senomorphics mainly refer to the inhibition of the SASP without damaging the senescent cells. Up to now, senolytics have demonstrated positive effects in mouse models presenting atherosclerosis, osteoarthritis, cataracts, cardiac hypertrophy, renal dysfunction, lipodystrophy, and sarcopenia [23, 25, 26, 27, 28].

More particularly, ovarian cancer cell lines (OVCAR3, OVCAR8, and OV90) showed decreased cell viability when treated with Talazoparib and Navitoclax (ABT‐263, a senolytic drug) compared to cells treated with Talazoparib or Navitoclax alone. The combination of Talazoparib and Navitoclax induced the accumulation of ovarian cancer cells in the sub‐G1 phase indicating an increase in DNA fragmentation. Furthermore, more apoptosis was observed in the presence of Talazoparib and Navitoclax compared to Talazoparib alone. The increased apoptosis was demonstrated by an increase in the percentage of cells positive for annexin V, an increase in caspase 3/7 activity, an increase in cleaved PARP and an increase in the protein abundance of some members of the Bcl‐2 family [29]. Fleury et al. confirmed these results *in vitro* and *in vivo*. Indeed, human cell lines derived from the ascites of patients diagnosed with high‐grade serous epithelial ovarian cancer (OV4453 and OV1946) were injected into NOD rag gamma (NRG) mice. A lower tumor size was observed when the mice were treated with Olaparib and Navitoclax compared to Olaparib alone. Similar conclusions were drawn in NRG mice in which breast cancer MDA‐MB‐231 cells were injected in the flanks [11]. Interestingly, unlike Fleury et al., Yue et al. demonstrated that Niraparib only induced senescence in MCF7 cells which were harboring a mutation in BRCA1 [30]. As aforementioned, the understanding of senescence has mainly derived from studying non‐transformed fibroblasts and primary epithelial cells. However, numerous studies have demonstrated the importance of understanding how anticancer therapies can induce senescence in cancer cells, in order to better target these senescent cells to improve therapeutic success. In this work, we deciphered how PARPi induce senescence in cancer cell lines using five clinically relevant inhibitors: Veliparib, Rucaparib, Olaparib, Niraparib, and Talazoparib. We showed that these inhibitors induced a senescence phenotype in nonsmall cell lung carcinoma (NSCLC) cell lines. NSCLC cell lines were chosen as several clinical trials are using PARPi, either alone or in combination, to treat patients with lung cancer [31]. Interestingly, we showed that Talazoparib induced a stronger senescence phenotype compared to the other PARPi. Furthermore, our results suggest that the senescence phenotype is not linked to the ability of the PARPi to inhibit the formation of pADPr. However, we demonstrated that PARP1 is required for Talazoparib to induce senescence. Finally, we found that a second hit with the senolytic drug Navitoclax eliminates senescent cancer cells, thus enhancing total cell death.

## Materials and methods

### Cell lines and culture procedures

Human A549 NSCLC cells (RRID:CVCL_0023; ATCC‐LGC Standards, Molsheim, France) and human Calu‐1 NSCLC cells (RRID:CVCL_0608; received from DKFZ Heidelberg) were subcultured in Glutamax Modified Eagle's Medium (Gibco Life Technologies) supplemented with 10% fetal bovine serum (FBS) (Gibco Life Technologies, Carlsbad, CA, USA). Human H460 NSCLC cells (RRID:CVCL_0459; received from DKFZ Heidelberg) were subcultured in Roswell Park Memorial Institute 1640 (RPMI 1640) (Gibco Life Technologies) supplemented with 10% FBS. For the experiments, the medium was supplemented with 0.5% penicillin/streptomycin (Sigma, Saint Louis, MO, USA). Cell lines were maintained at 37°C in 5% CO_2_. All cell lines were authenticated by genetic characteristics determined by PCR‐single‐locus‐technology (Eurofins Genomics, Ebersberg, Germany). They were tested negative for mycoplasma using MycoAlert™ Mycoplasma Detection Kit (Lonza, Rhisne, Belgium).

### Reagents

The following reagents were used: Olaparib (AZD2281, S1060; Selleckchem, Houston, TX, USA), Talazoparib (BMN673, S07048; Selleckchem), Niraparib (MK‐4827, S2741; Selleckchem), Rucaparib (AG‐014699, S1098; Selleckchem), Veliparib (ABT‐888, S1004; Selleckchem), and Navitoclax (ABT‐263, HY‐10087; MedChemExpress, Monmouth Junction, NJ, USA).

### Cell proliferation

To evaluate cell proliferation, cells were incubated with PARPi. Cell number was counted manually using trypan blue to exclude dead cells over a period of 6 days.

### Immunoblot analysis

Cells were lyzed in NP‐40 buffer (Tris–HCl 50 mM pH 7.4, NaCl 150 mM, EDTA 1 mM, NP‐40.1%) containing protease inhibitors and phosphatase inhibitors. The lysate was then centrifuged 15 min at 15 000 rpm at 4 °C and the supernatant was collected. 10–15 μg of proteins was separated on home‐made gel, and then, transferred onto a low fluorescence background polyvinylidene fluoride (PVDF) membrane (Millipore) using the wet blotting procedure. Membranes were blocked with Odyssey blocking buffer (Li‐Cor Biosciences, Lincoln, NE, USA) for 1 h at room temperature. Primary antibody against full‐length PARP1 (#9542; Cell Signaling, Danvers, MA, USA), pADPr (ab14459; Abcam, Cambridge, UK) were used at 1/1.000 and GAPDH (ab8245; Abcam) at 1/10.000. Anti‐mouse secondary antibody, IRDye 680RD and IRDye 800CW Goat anti‐mouse IgG (Li‐Cor Biosiences) and anti‐rabbit secondary antibody, IRDye 680RD and IRDye 800CW Goat anti‐rabbit IgG (Li‐Cor Biosciences) were used at 1/10.000. The membranes were scanned with the Li‐Cor Odyssey Infrared Imager (Li‐Cor Biosciences).

### Senescence‐associated beta‐galactosidase

Detection of senescence‐associated beta‐galactosidase was described in [32]. Briefly, cells were fixed with 2% formaldehyde and 0.2% glutaraldehyde diluted in PBS for 5 min, rinsed with PBS and with phosphate buffer (pH 5.8), and incubated at 37 °C, in a dry incubator, for 18 h, in the staining solution containing: 5‐bromo‐4‐chloro‐3‐inolyl‐β‐galactopyranoside in dimethylformamide (20 mg·mL^−1^) (#0428‐1G; Amresco, Radnor, PA, USA), phosphate buffer (pH 5.8), potassium ferricyanide (100 mm), potassium ferrocyanide (100 mm), NaCl (2.5 m), and MgCl_2_ (1 m). After the incubation, cells were rinsed twice with PBS and with methanol before observation under optical microscope for quantification. At least, 200 cells per well were counted.

### RNA extraction and RT‐qPCR

Total RNA was isolated from cells with ReliaPrep™ RNA Tissue Miniprep System (Z6111; Promega, Madison, WI, USA) according to the manufacturer's instructions. RNA concentrations were quantified using the Nanophotometer N60 (Implen, Munich, Germany). cDNA was synthesized with GoScript™ Reverse Transcription Mix (A2790; Promega) according to the manufacturer's instructions using random primers. 0.5–2 μg of RNA was used per reaction. The primers used for the qPCR are described in Table 1. A linear relationship between Ct values and cDNA concentration expressed in log_2_ was checked for all primer pairs. All real‐time PCR reactions were performed using 3 ng of cDNA. Reactions were performed in duplicates with GoTaq™ qPCR Master Mix (A6001; Promega) on a ViiA 7 Real‐Time PCR System (Applied Biosystems, Carlsbad, CA, USA) using a standard run. The gene expression level of each messenger RNA (mRNA) was calculated, further normalized to glyceraldehyde 3‐phosphate dehydrogenase enzyme (GAPDH) mRNA, and related to the control condition. Specific amplification was confirmed by melting curve analysis.

**Table 1:** Primer sequences.

Gene	Forward sequence (5′‐3′)	Reverse sequence (5′‐3′)
PARP1	GGGAGGGTCTGATGATAGC	CTGTCAACCACCTTAATGTCAG
CDKN1A	GTGGACCTGTCACTGTCTTG	GGCGTTTGGAGTGGTAGAAA
MKI67	AGAAGACAGTACCGCAGATGA	CGCCTCACTAATTTAACGCTGG
LMNB1	ACTGGCGAAGATGTGAAGGTTAT	CCCTGCTGGTGGAAAAGTTC
CCL2	AAGTGTCCCAAAGAAGCTGT	TGGGTTGTGGAGTGAGTGTT
CCL5	AGCCTCTCCCACAGGTACCAT	GCGGGCAATGTAGGCAAA
CXCL10	CCAGTCTCAGCACCATGAATC	GAGGTACTCCTTGAATGCCACT
IL6	CCTGAACCTTCCAAAGATGGC	CACCAGGCAAGTCTCCTCATT
IL8	CTGGCCGTGGCTCTCTTG	GGGTGGAAAGGTTTGGAGTATG
GAPDH	TGAAGGTCGGAGTCAACGG	GCAACAATATCCACTTTACCAGAGT

### Nucleus labeling

Cells were seeded on coverslips and fixed with 4% paraformaldehyde (PFA). The nuclei were stained with 2.5 mg·mL^−1^ DAPI (Sigma) for 10 min. Following PBS washes, the coverslips were mounted on microscope slides with Mowiol. The observations were performed by confocal microscopy by keeping the photomultiplier at a constant gain (Leica SP5, Leica Microsystems, Wetzlar, Germany). The nuclear area was determined using imagej.

### 5‐ethynyl 2′‐deoxyuridine (EdU) assay

Cells were seeded on coverslips in a 24‐well plate at a density of 8000 cells per well. Talazoparib (1 μm) was added 24 h later (Day 0) and Navitoclax (1 μm) a further 24 h later (day 1). On Day 3, the cells were incubated overnight in complete medium containing EdU at 10 μm (Baseclick, Munich, Germany). Cells were fixed with 4% PFA for 10 min, permeabilized with PBS + 1% bovine serum albumin (BSA) + 0.02% saponin during 30 min, incubated with the click assay cocktail following the manufacturer's instructions, washed with PBS + 1% BSA + 0.02% saponin and incubated with DAPI for 1 h. Cells were finally rinsed with PBS and coverslips were mounted with Fluoromount‐G. Fluorescent labellings were observed with confocal LSM900 microscope (Zeiss, Oberkochen, Germany).

### MTT assay

Cell viability was determined using MTT (3‐[4,5‐dimethylthiazol‐2‐yl]‐2,5‐diphenyltetrazolium bromide) (Sigma). Briefly, MTT was diluted in PBS at 2.5 mg·mL^−1^ and added to the cell media at a ratio of 1 : 1 for 2 h at 37 °C. After incubation, cells were lysed with dimethyl sulfoxide (DMSO) (Sigma). The optical density was read using a spectrophotometer at 570 nm.

### Generation of knock‐out PARP1 cell line

To generate A549 cells knock‐out for PARP1, two crRNAs were used (Table 2). crRNA (Integrated DNA Technologies [IDT], Coralville, IA, USA) was annealed to tracrRNA (IDT) using a thermocycler. In order to form the ribonucleoprotein (RNP) complex, Cas9 (IDT) was added to the crRNA:tracrRNA duplex. Approximately 1 × 10^6^ cells were harvested and resuspended in Nucleofector solution (IDT) supplemented with the RNP complex. The suspension was then placed in an Axama cuvette nucleocuvette for electroporation using program C‐09 (Amaxa, London, Germany). Cells were then plated in a 6‐wells plate. 24 h after electroporation, the medium was changed. 48 h after electroporation, the cells were placed in a 96‐wells plate for limit dilution. The knock‐out clones were identified via Sanger sequencing and immunoblot.

**Table 2:** crRNA sequences.

crRNA	Forward sequence (5′‐3′)
PARP1 (exon 1)	CGAGTCGAGTACGCCAAGAG
PARP1 (exon 11)	AACTCGGGGGGAAGTTGACG

### Statistical analyses

Statistical analyses were performed with GraphPad Prism. The unpaired two‐tail Student's *t*‐test was applied considering that the data were normally distributed. If the variance between groups was not similar, Welch's correction was applied. ANOVA I was applied considering that the data were normally distributed. If the variance between groups was not similar, the Kruskal–Wallis test was performed. The number of independent replicates for each experiment is indicated in the corresponding figure legend.

### Data availability

All primary data, including raw and unprocessed data and images, are available upon request.

## Results

### Talazoparib induces cellular senescence in non‐small cell lung carcinoma cell lines

**Fig. 1:**
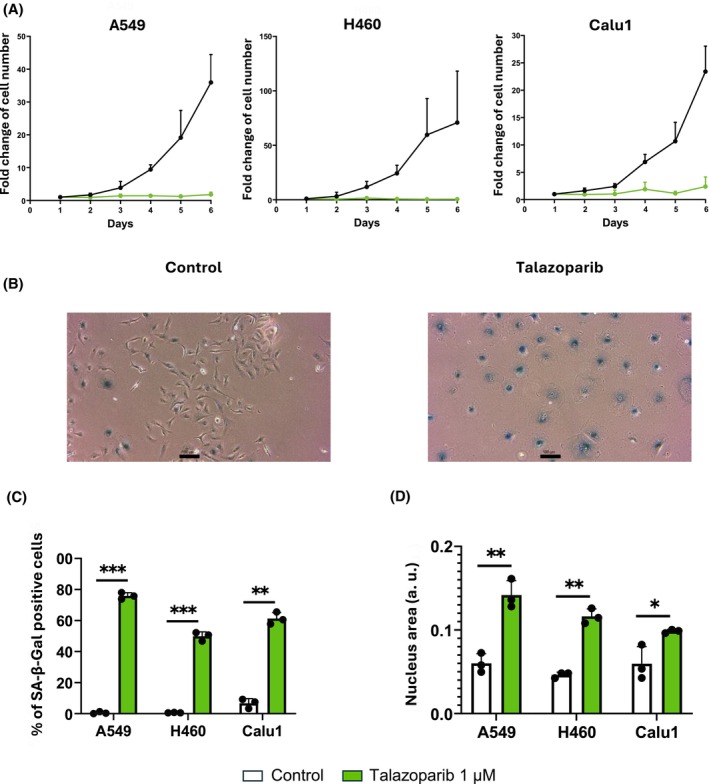
Talazoparib induces senescence in nonsmall cell lung carcinoma cell lines. (A) Proliferation arrest in A549, H460 and Calu1 cells incubated for 6 days with DMSO (control), Talazoparib 1 μm. Data are shown as mean ± 1 S.D. At least, 3 independent experiments were performed. (B) Representative pictures of SA‐b‐gal staining for A549 cells. Bar scale = 100 μm. (C) Percentage of SA‐β‐Gal positive cells for A549, H460 and Calu1 cells incubated for 6 days with DMSO (control), Talazoparib 1 μm. Data are shown as mean ± 1 S.D. At least, 3 independent experiments were performed. **P < 0.01 and ***P < 0.001, Unpaired *t*‐tests. (D) Nuclear area for A549, H460 and Calu1 cells incubated for 6 days with DMSO (control), Talazoparib 1 μm. Data are shown as mean ± 1 S.D. At least, three independent experiments were performed. *P < 0.01 and **P < 0.01, Unpaired *t*‐tests.

In a previous work, we investigated the induction of senescence in human A549 NSCLC cells treated with radiotherapy or radiotherapy combined to Olaparib, a PARPi [33]. Here, we first assessed if Talazoparib could also induce senescence in A549 cells. These cells were incubated with 1 μm of Talazoparib for 6 days. Treated cells showed a strong decrease in cell proliferation (Fig. 1A). As aforementioned, senescent cells are characterized by several features such as morphological changes, cell cycle arrest, increased lysosomal mass and the secretion of specific cytokines and chemokines. Senescence markers were observed in cancer cells after 6 days. A549 cells demonstrated an increase in the percentage of senescence‐associated beta‐galactosidase (SA‐β‐Gal)‐positive cells after treatment with 1 μm of Talazoparib (Fig. 1B). For A549 cells, the percentage was 76% for cells incubated with Talazoparib 1 μm compared to control cells exhibiting a negligible percentage of SA‐β‐Gal‐positive cells (Fig. 1C). In addition, other NSCLC cell lines, namely Calu1 and H460, were used to confirm that treatment with Talazoparib induced a senescence phenotype. Similar results were observed. Indeed, Talazoparib strongly reduced cell proliferation (Fig. 1A) and increased the percentage of SA‐β‐Gal‐positive cells in Calu1 and H460 cells (Fig. 1B). Interestingly, the percentage of SA‐β‐Gal‐positive cells after treatment with 1 μm of Talazoparib was 50% in H460 cells, and the percentage of SA‐β‐Gal positive cells was negligible in control cells. Calu1 cells showed 61% of SA‐β‐Gal‐positive cells after incubation with 1 μm of Talazoparib in comparison; the percentage of SA‐β‐Gal positive cells was around 10% in the control cells.

**Fig. 2:**
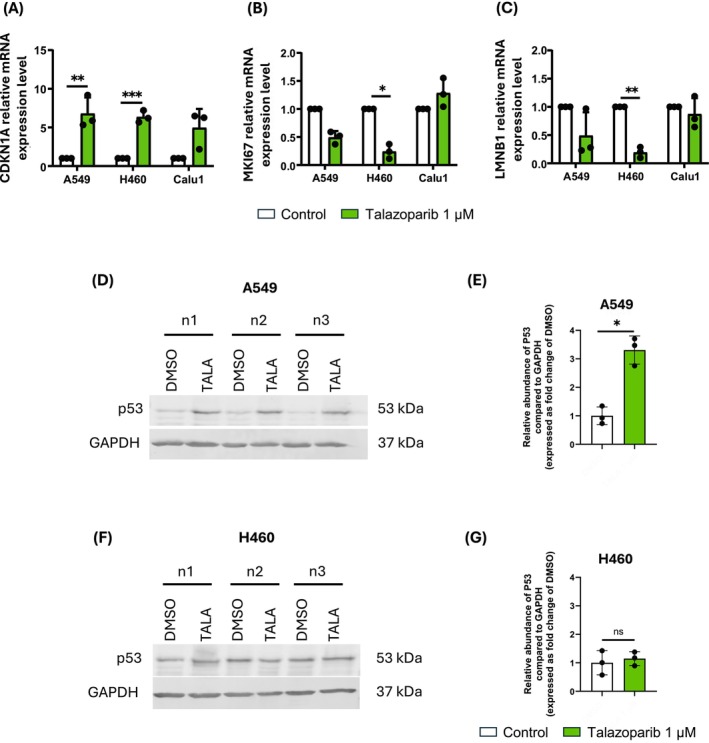
Talazoparib induces senescence in nonsmall cell lung carcinoma cell lines. (A) CDKN1A relative mRNA expression level, (B) MKI67 relative mRNA expression level and (C) LMNB1 relative mRNA expression level in A549, H460 and Calu1 cells incubated for 6 days with DMSO (control), Talazoparib 1 μm. Data are shown as mean ± 1 S.D. At least, 3 independent experiments were performed. *P < 0.01, **P < 0.01 and ***P < 0.001, Unpaired *t*‐tests. (D) Western blot analysis of the protein abundance of p53 relative to GAPDH as a loading control for A549 cells incubated for 6 days with DMSO (control), Talazoparib 1 μm, and quantified in (E). Data are shown as mean ± 1 S.D. for three independent experiments. *P* < 0.01, Unpaired *t*‐tests. (F) Western blot analysis of the protein abundance of p53 relative to GAPDH as a loading control for H460 cells incubated for 6 days with DMSO (control), Talazoparib 1 μm, and quantified in (G). Data are shown as mean ± 1 S.D. for three independent experiments.

**Fig. 3:**
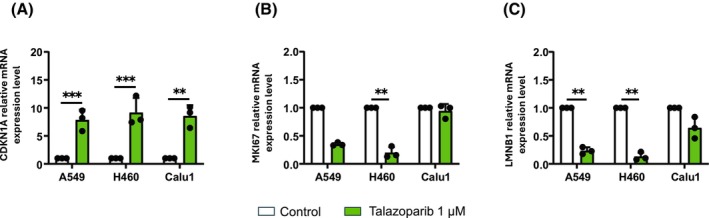
Talazoparib induces a senescence phenotype in nonsmall cell lung carcinoma cell lines. (A) CDKN1A, (B) LMNB1 and (C) MKI67 relative mRNA expression level in A549, H460 and Calu1 cells incubated for 3 days with DMSO (control), Talazoparib 1 μm. Data are shown as mean ± 1 S.D. At least, three independent experiments were performed. **P < 0.01 and ***P < 0.001, Unpaired *t*‐tests.

In addition, the shape of cells was modified as demonstrated by a significant increase in nuclear area observed after incubation with 1 μm of Talazoparib in A549, H460, and Calu1 cells (Fig. 1D). All three NSCLC cell lines also demonstrated a significant increase in cyclin dependent kinase inhibitor 1 (*CDKN1A*) expression at the mRNA level after three and 6 days of incubation with Talazoparib (Figs 2A and 3A). The CDKN1A gene encodes a cyclin‐dependent kinase inhibitor that can inhibit the activity of CDK2, CDK3, CDK4, and CDK6. The function of the *CDKN1A* gene is thus to act as a regulator of the cell cycle. Reduced proliferation following three or 6 days of Talazoparib treatment was also evidenced by the significant decrease in *MKI67* mRNA level in H460 cells (Figs 2B and 3B). Indeed, *MKI67* is described as being only present in proliferating cells [34]. The expression of *LMNB1* was significantly decreased following three or 6 days of incubation with Talazoparib in H460 cells at mRNA level (Figs 2C and 3C). This decreased *LMNB1* expression was observed in senescent cells, suggesting a rupture of the nuclear membrane, a known hallmark of cellular senescence [16]. Interestingly, the decrease in MKI67 and LMNB1 mRNA expression was not observed in Calu1 cells following incubation with Talazoparib. The absence of a decreased expression of *LMNB1* and *MKI67*, two senescence markers, in Calu1 cells might be linked to the lack of TP53 gene (homozygous deletion) [35], while A549 and H460 cells are wild‐type for TP53 [35, 36]. Furthermore, the abundance of p53 was checked in A549 and H460 cells to understand whether p53 was responsible for the increase in *CDKN1A* observed at mRNA level. Interestingly, the abundance of p53 increased in A549 cells following treatment with Talazoparib (Fig. 2D,E). However, in H460 cells, treatment with Talazoparib did not increase the abundance of p53 (Fig. 2F,G). This result indicates that cells may respond in a different way to PARP inhibition.

Thus, we concluded that Talazoparib induces a senescent phenotype in non‐small cell lung carcinoma cell lines characterized by proliferation arrest, an increase in the percentage of SA‐β‐Gal‐positive cells, and a significant increase in nuclear area.

### Cellular senescence is differentially induced by different PARP inhibitors

**Fig. 4:**
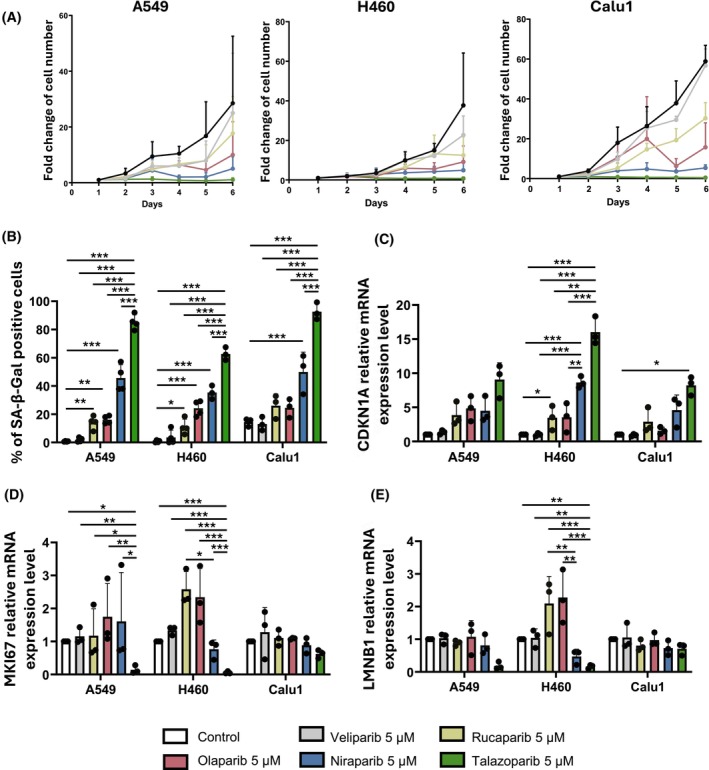
Induction of senescence by the different PARP inhibitors. (A) Proliferation arrest in A549, H460 and Calu1 cells incubated for 6 days with DMSO (control), Talazoparib 1 μm. Data are shown as mean ± 1 S.D. (B) Percentage of SA‐β‐Gal positive cells in A549, H460 and Calu1 cells incubated for 6 days with DMSO (control), Veliparib 5 μm, Rucaparib 5 μm, Olaparib 5 μm, Niraparib 5 μm or Talazoparib 5 μm. (C) CDKN1A relative mRNA expression level, (D) LMNB1 relative mRNA expression level and (E) MKI67 relative mRNA expression level in A549, H460 and Calu1 cells incubated for 6 days with DMSO (control), Veliparib 5 μm, Rucaparib 5 μm, Olaparib 5 μm, Niraparib 5 μm or Talazoparib 5 μm. Data are shown as mean ± 1 S.D. At least, three independent experiments were performed. *P < 0.01, **P < 0.01 and ***P < 0.001. ANOVA I.

**Fig. 5:**
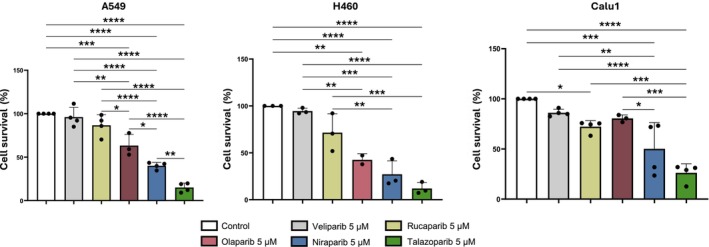
The inhibitors of PARP do not induce the same level of cell death. Cell survival evaluated via MTT of A549, H460 and Calu1 cells incubated for 6 days with DMSO (control), Veliparib 5 μm, Rucaparib 5 μm, Olaparib 5 μm, Niraparib 5 μm or Talazoparib 5 μm. Data are shown as mean ± 1 S.D. At least, three independent experiments were performed. *P < 0.01, **P < 0.01, ***P < 0.001 and *P* < 0.0001. ANOVA I.

**Fig. 6:**
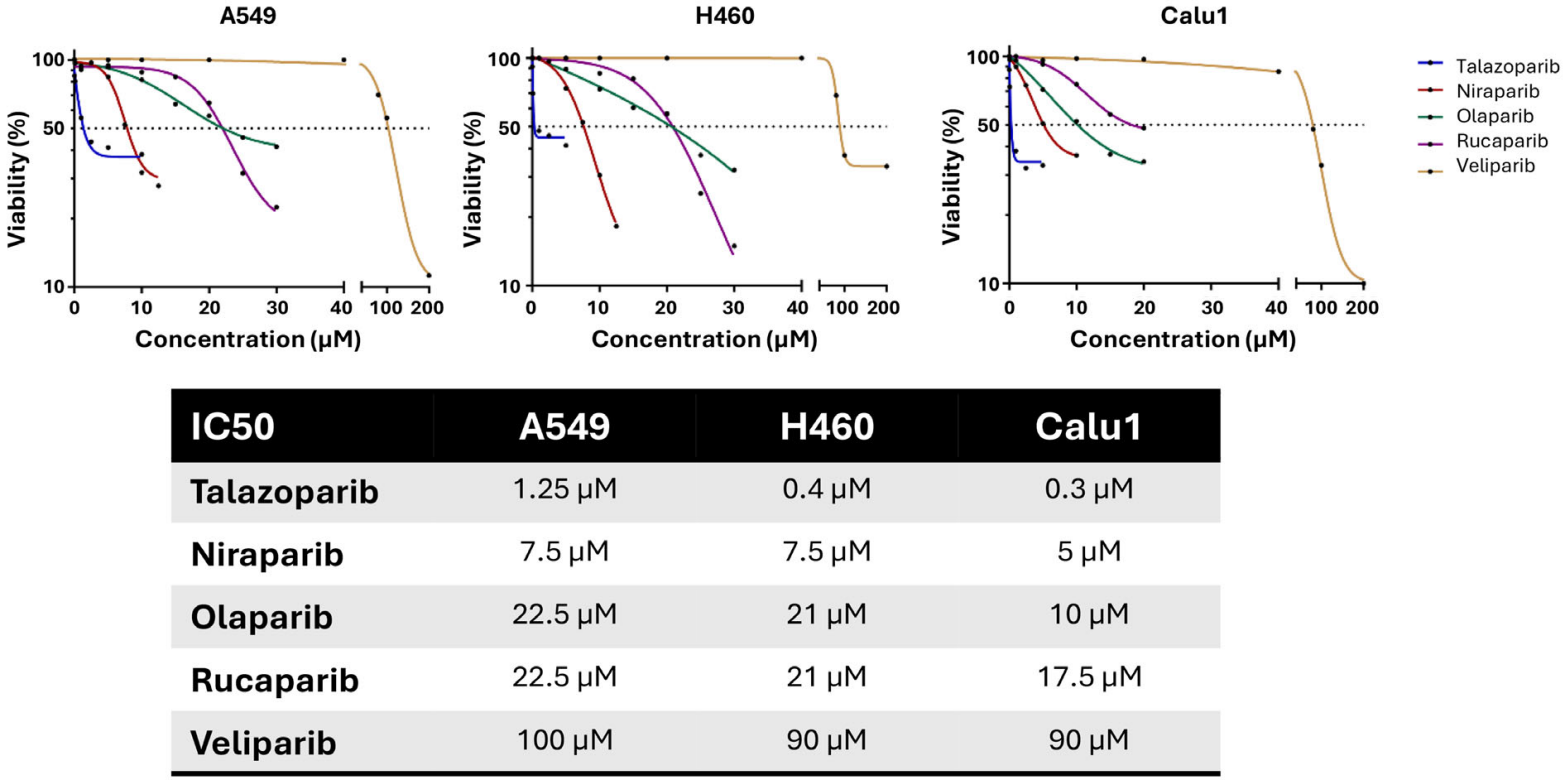
Determination of the IC50 of the different PARP inhibitors in A549, H460, and Calu1 cells. Cell survival evaluated via MTT of A549, H460, and Calu1 cells incubated for 6 days with DMSO (control) or different concentrations of the PARPi.

To better understand how cellular senescence was induced following treatment with PARPi, five inhibitors were compared: Talazoparib, Niraparib, Olaparib, Rucaparib, and Veliparib. NSCLC cells were incubated with 5 μm of Talazoparib, Niraparib, Olaparib, Rucaparib, or Veliparib. Cell proliferation was impacted in all three cell lines (Fig. 4A). The strongest impact on cell proliferation was observed in cells incubated with Talazoparib or with Niraparib. Rucaparib and Olaparib induced a similar moderate inhibition of cell proliferation, whereas Veliparib only slightly affected cell proliferation. Furthermore, Niraparib and Talazoparib were also the most toxic molecules in three cell lines as determined by a MTT assay (Figs 5 and 6).

The proportion of SA‐β‐Gal‐positive cells was then assessed following incubation with the different PARPi for 6 days. The percentage of SA‐β‐Gal‐positive cells was significantly increased in A549 and H460 cells incubated with Rucaparib, Olaparib, Niraparib, or Talazoparib. For Calu1 cells, only Niraparib and Talazoparib significantly increased the proportion of SA‐β‐Gal positive cells (Fig. 4B). Furthermore, the percentage of SA‐β‐Gal positive cells was significantly higher when A549, H460, and Calu1 cells were incubated with 5 μm of Talazoparib compared to the other PARPi.

**Fig. 7:**
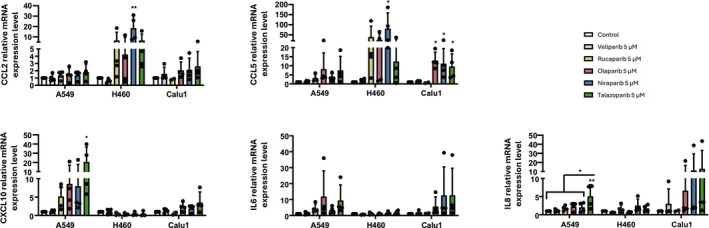
Induction of SASP by the different PARP inhibitors. (A) CCL2, (B) CCL5, (C) CXCL10, (D) IL6, and (E) IL8 relative mRNA expression level in A549, H460 and Calu1 cells incubated for 6 days with DMSO (control), Veliparib 5 μm, Rucaparib 5 μm, Olaparib 5 μm, Niraparib 5 μm or Talazoparib 5 μm. Data are shown as mean ± 1 S.D. At least, three independent experiments were performed. *P < 0.01 and **P < 0.01. ANOVA I.

After 6 days, all three cell lines were also analyzed for their expression of *CDKN1A* at the mRNA level (Fig. 4C). *CDKN1A* expression significantly increased in H460 and Calu1 cells incubated with 5 μm of Talazoparib when compared to control cells. In H460 cells, Talazoparib significantly increased *CDKN1A* expression when compared to Veliparib, Rucaparib, and Olaparib. The expression of *MKI67* was significantly decreased in A549 and H460 cells incubated with Talazoparib compared to control cells (Fig. 3D). The expression of *LMNB1* was significantly decreased in H460 cells incubated with Talazoparib compared to control cells and to other PARPi (Fig. 4E). Since the SASP can have protumoral or antitumoral effects, several genes were characterized in A549, H460, and Calu1 cells. The expression of CCL2, CCL5, CXCL10, IL6, and IL8 was evaluated in A549, H460, and Calu1 cells after treatment with PARPi; the expression is heterogeneous among these three cell lines (Fig. 7). These results demonstrated for the first time that inhibitors of PARP are not all equal in the induction of senescence.

### The inhibition of PARP1 catalytic activity by the different inhibitors is similar

**Fig. 8:**
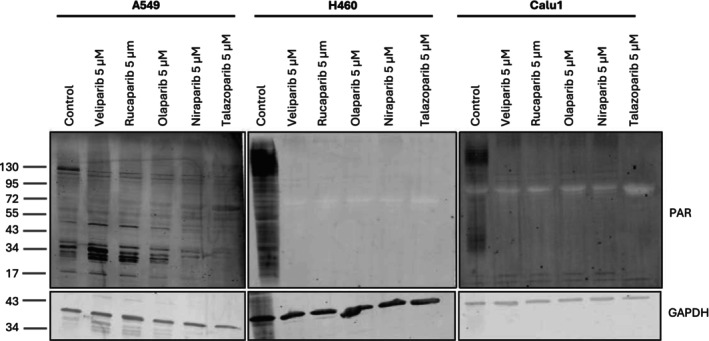
The catalytic inhibition of PARP is similar for the different inhibitors. Immunoblot for PARylated proteins in A549, H460, and Calu1 cells incubated for 6 days with DMSO (control), Veliparib 5 μm, Rucaparib 5 μm, Olaparib 5 μm, Niraparib 5 μm, or Talazoparib 5 μm. GAPDH was used as a loading control. Representative blot of three biological replicates.

To define whether the inhibition of PARP1 catalytic activity is responsible for the senescence phenotype, the formation of PAR/pADPr was assessed in cells incubated or not with each of the PARPi. We showed that the five PARPi at 6 days completely abrogated the formation of pADPr in H460 and Calu1 cancer cells, while in A549 cells, Veliparib and Rucaparib were less efficient to inhibit PARylation of lower molecular weight proteins (Fig. 8). This result indicates that the catalytic inhibition is not responsible for the differential induction, according to the inhibitor, of the senescence phenotype observed in NSCLC cells.

### The presence of PARP1 is required for the induction of senescence

**Fig. 9:**
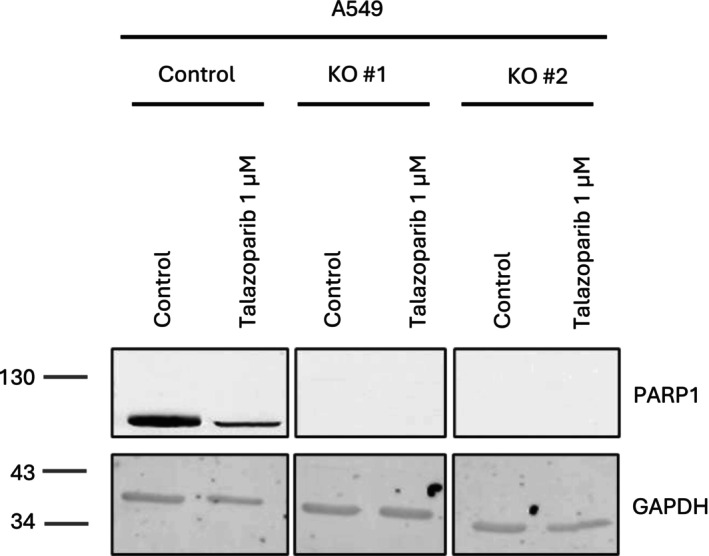
Validation of the knock‐out for PARP1 in individual clones. Immunoblot of PARP1 of the chosen CRISPR/Cas9 clones incubated for 6 days with DMSO (control) or Talazoparib 1 μm. GAPDH was used as a loading control. Three independent biological replicates were performed.

**Fig. 10:**
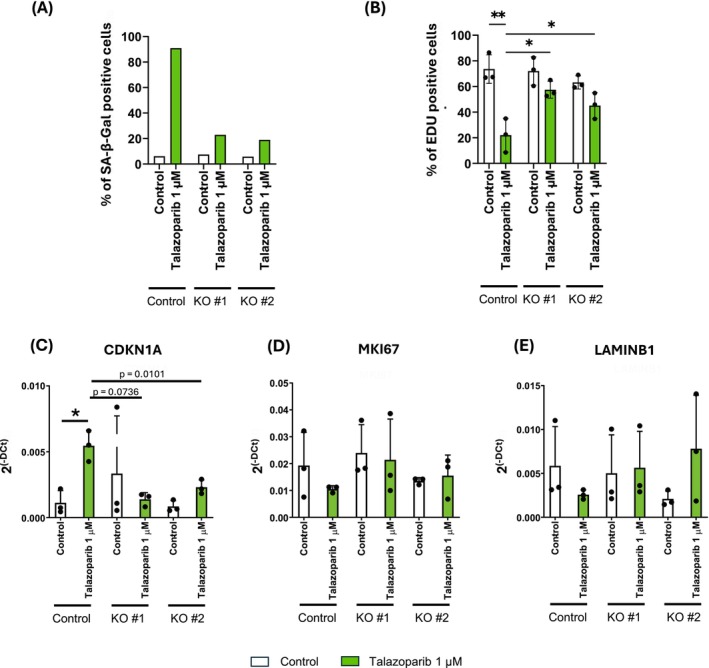
PARP1 is necessary for the senescence phenotype to occur in A549 cancer cell line. (A) Percentage of SA‐β‐Gal‐positive cells in A549 CRISPR/Cas9 clones incubated for 6 days with DMSO (control), Talazoparib 1 μm. One biological replicated was performed. (B) Quantification of the percentage of EdU‐positive cells. A total of 100 cells were counted for each condition. 3 independent biological replicates were performed. Data are shown as mean ± 1 S.D. *P < 0.01. ANOVA (C) CDKN1A relative mRNA expression level, (D) MKI67 relative mRNA expression level and (E) LMNB1 relative mRNA expression level in A549 CRISPR/Cas9 clones incubated for 6 days with DMSO (control), Talazoparib 1 μm. Three independent biological replicates were performed. Data are shown as mean ± 1 S.D. *P < 0.01. ANOVA I.

To determine whether the absence of PARP1 would lead to senescence similar to that observed with some PARP inhibitors, a CRISPR/Cas9 genome editing method was used to introduce short frameshift insertions‐deletions (indels) in the PARP1 gene, creating a PARP1 knock‐out. Two single‐guide RNA (sgRNAs) were designed, targeting exon 1 and exon 11 of the PARP1 gene. Several clones were isolated. For subsequent analyses, one wild‐type clone and two knock‐out clones were used. The clones were validated using Sanger sequencing and immunoblot. This experiment was performed using A549 cells (Fig. 9). Interestingly, treatment with 1 μm Talazoparib appears to affect the abundance of PARP1 protein. We hypothesized that it is due to the absence of pADPr as it is known that PARP1 is capable of modifying itself. Indeed, this automodification is essential for its function in DNA repair, as it allows PARP1 to assemble into liquid‐like structures, recruit other repair proteins. It also seems to favor the stability of the protein [37, 38]. When the wild‐type cells were exposed to Talazoparib (1 μm), the percentage of cells positive for SA‐β‐Gal significantly increased (Fig. 10A). This effect was accompanied by a significant decrease in the number of proliferating cells as shown by the decrease in the number of EDU‐positive cells (Fig. 10B). Moreover, the expression of CDKN1A was also increased (Fig. 9C) whereas the expression of *MKI67* and *LMNB1* was decreased at the mRNA level (Fig. 10D,E). These results confirmed the previously obtained results in A549 cells. Interestingly, the percentage of SA‐β‐Gal positive cells in non‐exposed knock‐out cells was similar to the percentage observed for the wild‐type cells indicating that the absence of PARP1 did not induce senescence *per se* in A549 cells (Fig. 10A). Furthermore, when the knock‐out cells were exposed to Talazoparib (1 μm), the percentage of SA‐β‐Gal positive cells was strongly reduced compared to the wild‐type cells (Fig. 10A) and the decrease in the number of proliferating cells was smaller (Fig. 10B). The increase in the expression of *CDKN1A* at mRNA level was also reduced in the cells of the two knock‐out clones (Fig. 10C). The decrease in the expression of *MKI67* and *LMNB1* mRNA was reduced in knock‐out clones compared to the wild‐type clone (Fig. 10D,E).

### Navitoclax can synergize with Talazoparib in the presence of PARP1

**Fig. 11:**
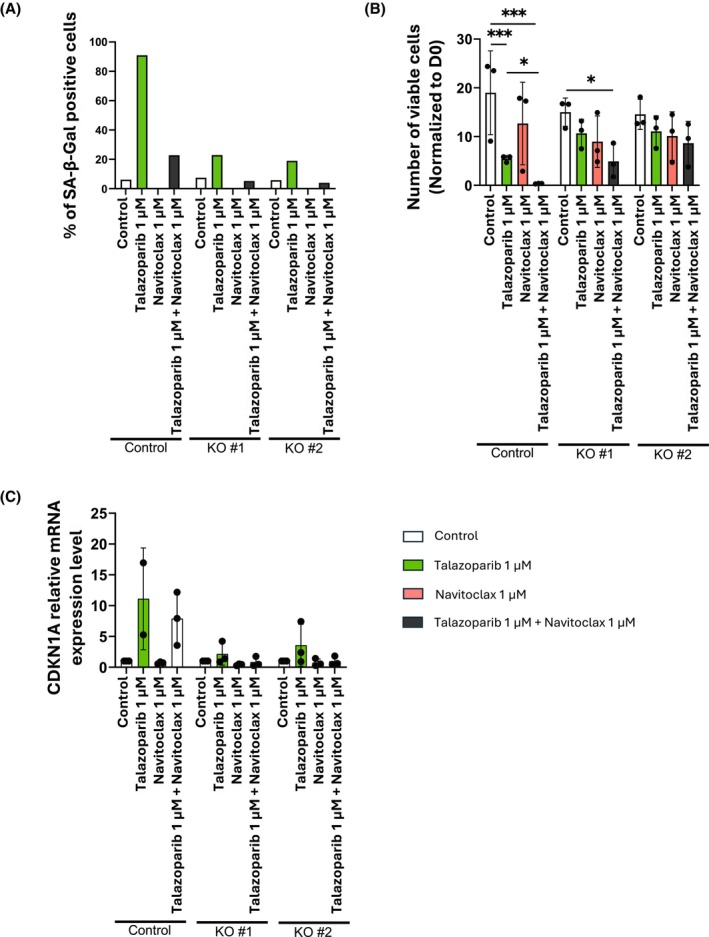
PARPi‐induced senescent cells are eliminated by Navitoclax. (A) Percentage of SA‐β‐Gal positive cells in A549 CRISPR/Cas9 clones incubated for 6 days with DMSO (control), Talazoparib 1 μm with or without Navitoclax 1 μm. One biological replicate was performed. (B) A549 CRISPR/Cas9 clones incubated for 6 days with DMSO (control), Talazoparib 1 μm with or without Navitoclax 1 μm. and cell survival was evaluated via MTT. Three independent biological replicates were performed. Data are shown as mean + S.D. *P < 0.01. ANOVA (C) CDKN1A relative mRNA expression level in A549 CRISPR/Cas9 clones incubated for 3 days with DMSO (control), Talazoparib 1 μm with or without Navitoclax 1 μm. Three independent biological replicates were performed. Data are shown as mean ± 1 S.D.

To determine whether Navitoclax can induce cell death in the cells treated with Talazoparib, wild‐type and knock‐out for PARP1 cells were treated with both Talazoparib and Navitoclax. A strong reduction in the percentage of SA‐β‐Gal‐positive cells was observed in cells treated with both Talazoparib and Navitoclax compared to cells treated with Talazoparib alone (Fig. 11A). Indeed, in wild‐type cells treated with Talazoparib alone, the percentage of SA‐β‐Gal‐positive cells was 90% while in wild‐type cells treated with both Talazoparib and Navitoclax, the percentage of SA‐β‐Gal‐positive cells was 22%. Moreover, in knock‐out cells, the percentage of SA‐β‐Gal‐positive cells treated with Talazoparib was approximately 20% while in knock‐out cells treated with both Talazoparib and Navitoclax, the percentage of SA‐β‐Gal positive cells further decreased below 5%. The results are in line with the number of viable cells observed after treatment with both Talazoparib and Navitoclax. Indeed, the number of viable cells was also significantly reduced when wild‐type cells were treated with both Talazoparib and Navitoclax compared to Talazoparib alone (Fig. 11B). In the presence of Talazoparib alone, the number of viable wild‐type cells was equivalent to 5, while in the presence of Talazoparib and Navitoclax, the number of viable wild‐type cells decreased to less than 1. In the knock‐out cells, the number of viable cells was approximately 10 in the presence of Talazoparib alone, whereas the number of viable cells in the presence of Talazoparib and Navitoclax was approximately 6. As aforementioned, the expression of CDKN1A was also reduced in knock‐out cells treated with Talazoparib compared to wild‐type cells treated with Talazoparib. Furthermore, wild‐type cells treated with both Talazoparib and Navitoclax demonstrated lower CDKN1A mRNA expression compared to cells treated with Talazoparib alone. The same trend was also observed for knock‐out cells (Fig. 11C).

Altogether, these results indicate that the induction of cellular senescence following PARPi treatment is at least partly dependent on the presence of PARP1.

## Discussion

The purpose of this work was to define whether all the PARPi induce senescence to a similar extent in cancer cells. We showed that Talazoparib induced a stronger senescence phenotype in cancer cells compared to Niraparib, Olaparib, Rucaparib, and Veliparib. We demonstrated an increased senescence phenotype in three different NSCLC cell lines. Of note, A549 and H460 cancer cells are known as TP53 wild‐type, whereas Calu1 cancer cells lack TP53 (homozygous deletion). It thus seems that PARPi can induce a senescence phenotype in a p53‐independent manner. These results are in accordance with what was described by [11] in ovarian cancer cells [11]. The authors used four different cell lines, namely OV1369(R2), OV90, OV4453, and OV1946, all harboring TP53 mutations. Nonetheless, the four cell lines demonstrated a senescence phenotype following treatment with Olaparib. Interestingly, in our work, the reduced expression of *LMNB1* and *MKI67* following treatment with Talazoparib was not observed in Calu1 cells. These results might indicate that p53 is involved in the induction of some features of senescence. Furthermore, the abundance of p53 was increased in A549 cells, while the abundance of p53 did not increase in H460 cells. These results, thus, indicate that even in TP53 wild‐type, the mechanism responsible for inducing senescence vary. To better understand the implication of p53 in the acquisition of the senescence phenotype, it would be necessary to delete p53 in A549 cells and/or in H460 cells.

Furthermore, we showed that the percentage of SA‐β‐Gal‐positive cells was different in the different cell lines. A549 and Calu1 cells showed the strongest induction in the percentage of SA‐β‐Gal‐positive cells compared to H460 cells. Indeed, 85% of A549 cells and 92% of Calu1 cells were positive for SA‐β‐Gal following treatment with 5 μm of Talazoparib while 62% of H460 cells were positive for SA‐β‐Gal following treatment with 5 μm of Talazoparib. These differences could be explained by differences in sensitivity to Talazoparib treatment. Fleury et al. showed that low sensitivity to treatment measured by cell death led to a high induction of SA‐β‐Gal‐positive cells [11]. However, we showed that the percentage of cell death was very similar in A549, H460 and Calu1 cells incubated with 5 μm of Talazoparib for 6 days. The cell survival of A549, Calu1, and H460 cells after treatment with 5 μm of Talazoparib was 15%, 26%, and 12%, respectively. Hence, another mechanism might be responsible for this discrepancy. One hypothesis was recently unveiled by Lombard et al. Indeed, these authors observed that Olaparib induced senescence in several prostate cancer cell lines. However, some cells were capable of evading senescence via G2/M checkpoint override. Furthermore, the authors also showed that cells capable of evading senescence following treatment with PARPi did not show an increase in p21 expression. Thus, a second hypothesis is that the ability of cancer cells to undergo senescence might be associated with p21 expression [12].

We further demonstrated that inhibiting the catalytic site of PARP1 was not responsible for inducing the senescence phenotype. Indeed, the formation of pADPr was similar in the three cell lines treated with 5 μm of Veliparib, Rucaparib, Olaparib, Niraparib, or Talazoparib. Furthermore, we demonstrated that the absence of PARP1 by itself did not lead to senescence in A549 cells. Exposure of A549 cells KO for PARP1 to Talazoparib did not induce senescence. Indeed, the A549 cells KO for PARP1 following treatment with Talazoparib showed no increase in SA‐β‐Gal‐positive cells, unlike wild‐type A549 cells treated with Talazoparib. As mentioned earlier, PARPi exert their effects by inhibiting the PARP1 catalytic site, but also by causing the formation of PARP1‐DNA complexes. A hypothesis is that the senescence phenotype observed in NSCLC cell lines is linked to the formation of PARP1‐DNA complexes and not to PARP catalytic activity.

These results thus indicate that PARPi trigger a senescence phenotype in NSCLC cells but that not all PARPi are equal in the induction of cellular senescence. Indeed, Talazoparib was the strongest inducer of senescence among the PARP inhibitors that we tested in this study. With the strong interest focused on the use of senolytics in cancer, these results demonstrate the importance of characterizing the senescence phenotype induced by anticancer treatment. Furthermore, PARPi were shown to have an impact on normal cells as described by Kieronska‐Rudek A. et al., 2025. Indeed, the authors demonstrated that Olaparib increases several senescent markers in mouse macrophages [39]. We also investigated the effect of Talazoparib on a nontransformed cell line (HBEC3‐KT cells which are normal human bronchial epithelial cells); it was observed that Talazoparib also induces a senescent phenotype in the nontransformed cell line (data not shown). However, it remains to be determined whether all PARPi affect nontransformed cells, and to which extent this might affect the efficacy of the treatment.

Our results established that senolysis by Navitoclax is effective in sensitizing cancer cells to PARPi. These results are in line with previous studies performed using Navitoclax to eliminate senescent cells induced by other kinds of anticancer treatments, being radiotherapy or chemotherapy [33, 40, 41]. Senzitisation to PARPi has also been reported for triple negative breast cancer cells and in ovarian cancer cells [42]. Hence, the targeting of senescent cancer cells perhaps may enhance PARPi efficacy and reduce the potential for cancer relapse.

Our study has several limitations that should be addressed in future research. In particular, it remains unclear whether PARPi treatment induces senescence in NSCLC tumors. If such an effect is confirmed, it will be important to investigate the efficacy of Navitoclax in combination with PARPi in the context of NSCLC tumors.

In the end, deepening current understanding regarding senescence in the context of cancer might bring new therapeutic venues for cancer patients.

## Conflict of interest

The authors declare no potential conflict of interest.

## Author contributions

CH and MVDA designed, conducted and analyzed the experiments, interpreted the experimental data, created the figures and wrote the manuscript with the assistance from CS, KB, AS and, A.K. A‐CW and CM developed the concept, led and supervised the studies.

## Data Availability

The datasets underlying the article will be shared upon a reasonable request to the corresponding author.
